# Method for Monitoring the Safety of Urban Subway Infrastructure Along Subway Lines by Fusing Inter-Track InSAR Data

**DOI:** 10.3390/s26020454

**Published:** 2026-01-09

**Authors:** Guosheng Cai, Xiaoping Lu, Yao Lu, Zhengfang Lou, Baoquan Huang, Yaoyu Lu, Siyi Li, Bing Liu

**Affiliations:** 1 Key Laboratory of Spatio-Temporal Information and Ecological Restoration of Mines, Ministry of Natural Resources of the People’s Republic of China, Henan Polytechnic University, Jiaozuo 454003, China; 2 Land Satellite Remote Sensing Application Center, Ministry of Natural Resources, Beijing 100048, China

**Keywords:** surface subsidence, inter-track fusion, Radarsat-2, SBAS-InSAR, key infrastructure monitoring

## Abstract

Urban surface subsidence is primarily induced by intensive above-ground and underground construction activities and excessive groundwater extraction. Integrating InSAR techniques for safety monitoring of urban subway infrastructure is therefore of great significance for urban safety and sustainable development. However, single-track high-spatial-resolution SAR imagery is insufficient to achieve full coverage over large urban areas, and direct mosaicking of inter-track InSAR results may introduce systematic biases, thereby compromising the continuity and consistency of deformation fields at the regional scale. To address this issue, this study proposes an inter-track InSAR correction and mosaicking approach based on the mean vertical deformation difference within overlapping areas, aiming to mitigate the overall offset between deformation results derived from different tracks and to construct a spatially continuous urban surface deformation field. Based on the fused deformation results, subsidence characteristics along subway lines and in key urban infrastructures were further analyzed. The main urban area and the eastern and western new districts of Zhengzhou, a national central city in China, were selected as the study area. A total of 16 Radarsat-2 SAR scenes acquired from two tracks during 2022–2024, with a spatial resolution of 3 m, were processed using the SBAS-InSAR technique to retrieve surface deformation. The results indicate that the mean deformation rate difference in the overlapping areas between the two SAR tracks is approximately −5.54 mm/a. After applying the difference-constrained correction, the coefficient of determination (*R*^2^) between the mosaicked InSAR results and leveling observations increased to 0.739, while the MAE and RMSE decreased to 4.706 and 5.538 mm, respectively, demonstrating good stability in achieving inter-track consistency and continuous regional deformation representation. Analysis of the corrected InSAR results reveals that, during 2022–2024, areas exhibiting uplift and subsidence trends accounted for 37.6% and 62.4% of the study area, respectively, while the proportions of cumulative subsidence and uplift areas were 66.45% and 33.55%. In the main urban area, surface deformation rates are generally stable and predominantly within ±5 mm/a, whereas subsidence rates in the eastern new district are significantly higher than those in the main urban area and the western new district. Along subway lines, deformation rates are mainly within ±5 mm/a, with relatively larger deformation observed only in localized sections of the eastern segment of Line 1. Further analysis of typical zones along the subway corridors shows that densely built areas in the western part of the main urban area remain relatively stable, while building-concentrated areas in the eastern region exhibit a persistent relative subsidence trend. Overall, the results demonstrate that the proposed inter-track InSAR mosaicking method based on the mean deformation difference in overlapping areas can effectively support subsidence monitoring and spatial pattern identification along urban subway lines and key regions under relative calibration conditions, providing reliable remote sensing information for refined urban management and infrastructure risk assessment.

## 1. Introduction

In the process of rapid urbanization, excessive surface and underground construction, along with over-exploitation of groundwater resources, has led to urban ground subsidence, which has become one of the major geological hazards restricting sustainable urban development [1,2,3]. Therefore, using high-resolution Synthetic Aperture Radar (SAR) imagery to obtain temporal subsidence data and analyze the changing patterns in urban areas is of significant importance for ensuring urban safety and scientific planning [1].

Traditional surface deformation measurement methods include leveling [2], photogrammetry [3], laser scanning [4], and Global Navigation Satellite System (GNSS) technology [5]. However, these methods have many limitations, including high costs, long monitoring periods, susceptibility to adverse weather conditions, and difficulty in achieving continuous monitoring over large areas [6]. In contrast, Interferometric Synthetic Aperture Radar (InSAR) technology can utilize phase information from SAR imagery to obtain continuous deformation fields in the line-of-sight (LOS) direction, and has become an important technical method for monitoring geological hazards [7]. Commonly used InSAR techniques include Differential InSAR (D-InSAR) [8], Permanent Scatterer InSAR (PS-InSAR) [9], and Small Baseline Subset InSAR (SBAS-InSAR) [10]. D-InSAR is suitable for monitoring short-term large deformations, and has been applied in satellite-based D-InSAR studies on earthquake fault ruptures, thereby expanding the application boundaries of InSAR technology in the field of seismic geology [11]. Qiao Xuejun and others utilized InSAR technology to obtain a digital elevation model of the Three Gorges region, further demonstrating the enormous potential of this technology in high-precision topographic mapping [12]. With deeper research, the limitations of D-InSAR technology in long-term slow deformation monitoring have gradually become apparent, particularly with phase errors caused by atmospheric disturbances, which significantly affect the inversion accuracy of D-InSAR [13]. In contrast, PS-InSAR and SBAS-InSAR overcome issues such as temporal coherence loss and atmospheric delay interference, and have been widely applied in monitoring urban slow subsidence with millimeter-level accuracy [14,15]. Farina et al. combined PS-InSAR with aerial imagery and optical satellite images for landslide investigation, discovering additional landslide areas in the Arno River basin [16]. Heleno et al. used PS-InSAR to monitor ground subsidence in Lisbon, finding that the primary cause of subsidence was the excessive extraction of groundwater [17]. Tizzani et al. employed SBAS-InSAR to study typical topographic deformations in Eastern California, including the Long Valley Caldera and Mono Basin, and compared the analysis results with GPS and leveling data, verifying the accuracy of this method in long time-series surface deformation analysis [10]. Kim used SBAS-InSAR to obtain vertical displacement results and analyzed changes in the extent and rate of ground subsidence, noting that subsidence in the area was slowing, with some regions approaching stability, reflecting effective control of the urban groundwater decline trend [18]. Kulsoom et al. combined SBAS-InSAR deformation results with the XGBoost model to construct a landslide susceptibility map along the Karakoram Highway, expanding the application of SBAS-InSAR technology in geological disaster prediction [19]. Hou Anye and others compared and analyzed the ground subsidence monitoring results of PS-InSAR and SBAS-InSAR, finding that both methods provided highly consistent results with actual monitoring data in the Beijing area [20]. Sun Xiaopeng et al. used SBAS-InSAR to monitor post-earthquake surface deformation in Chengdu, finding an overall uplift trend and controlling data errors within 3 mm, proving the reliability of the SBAS-InSAR method [21]. Zhou Quan and others applied SBAS-InSAR to identify deformations from Shadong Township to Xiong Song Township, successfully detecting multiple landslide areas and providing technical support for geological disaster risk early warning [22].

With the improvement in satellite-based SAR resolution, InSAR technology based on high-resolution SAR imagery has significantly enhanced its monitoring capabilities in complex urban environments [23]. However, high-resolution imaging modes typically sacrifice swath width, meaning that a single scene cannot cover the entire urban area. To obtain complete deformation information, multiple SAR images need to be mosaicked [24]. However, SAR images from different orbits or modes differ in observation geometry, reference point stability, atmospheric delays, and noise distribution, and direct mosaicking often leads to distortion in the results, thereby reducing the accuracy of subsidence monitoring [25,26,27]. Therefore, effectively integrating and mosaicking multi-orbit InSAR data while ensuring the accuracy of single-scene InSAR remains a key scientific challenge for precise urban surface subsidence monitoring [28]. Previous studies have used medium-resolution SAR imagery from ERS-1/2, ENVISAT ASAR, JERS-1, and Radarsat-1 to monitor urban surface subsidence [29,30]. Since its launch in 2014, Sentinel-1 has provided abundant temporal data for periodic subsidence monitoring [31,32,33]. The second-generation high-resolution SAR systems (e.g., TerraSAR-X/TanDEM-X, COSMO-SkyMed, Radarsat-2, ALOS-1/2) have provided a refined data foundation for urban infrastructure monitoring [34,35,36]. These studies primarily focus on the effectiveness of InSAR technology in urban subsidence monitoring, without addressing the issue of mosaicking multi-orbit InSAR data. Therefore, this paper selects the urban area of Zhengzhou, Henan Province as the study area, using high-resolution Radarsat-2 SAR data as the data source, and employs the SBAS-InSAR technique to invert the surface deformation in the study area. Based on this, the surface subsidence patterns in the urban area are analyzed along subway lines and in typical regions, providing scientific evidence for urban geological disaster prevention and infrastructure safety.

## 2. Materials and Methods

### 2.1. Study Area

**Figure 1:**
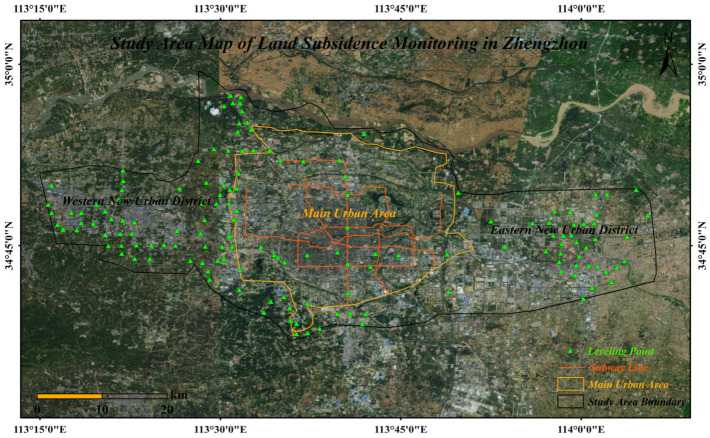
Study area.

Zhengzhou, the national central city, is located in the southern part of the North China Plain. It has a flat terrain and a temperate monsoon climate, with distinct seasons. The annual average precipitation is approximately 640 mm, and the annual average temperature is about 14.3 °C. In recent years, with the acceleration of urbanization, Zhengzhou has made significant progress in expanding its transportation hubs and new urban areas. Large-scale subway construction and major infrastructure projects have been continuously advancing, intensifying the risk of surface deformation. Based on this, this study selects the western new district, the main urban area (within the Fourth Ring), the area outside the Fourth Ring, and the eastern new district of Zhengzhou as the research area (113°15′–114°10′ E, 34°40′–35°00′ N). The research scope covers regions of Zhengzhou with high functional concentration, as shown in Figure 1. The study will focus on the spatiotemporal distribution characteristics of surface subsidence and deformation in key infrastructures such as transportation hubs, subway lines, and public facilities during the urbanization process, aiming to provide scientific support for urban planning and disaster prevention and mitigation.

### 2.2. Data

#### 2.2.1. SAR Data

Radarsat-2 is the next-generation C-band commercial synthetic aperture radar satellite launched by Canada, following Radarsat-1. It was launched on 14 December 2007, and operates in a sun-synchronous orbit at an altitude of approximately 798 km, with a revisit cycle of 24 days. The satellite offers multiple beam modes, supports both left and right side-view imaging, and provides SAR imagery with six different spatial resolutions: 1 m, 3 m, 5 m, 8 m, 25 m, and 30 m, to meet multi-scale monitoring needs. In this study, Radarsat-2 satellite HH polarization mode data in the ascending orbit direction is selected as the main data source for InSAR monitoring. The image spatial resolution is 3 m, with a swath width of 50 km × 50 km, making it suitable for high-precision deformation monitoring at the urban scale. Two SAR images are required for the study area, labeled as Image 1 and Image 2, with an incidence angle of 39.57° for Image 1 and 30.55° for Image 2. The SAR imagery information used is shown in Table 1.

#### 2.2.2. Optical Imagery Data

The JL-1 satellite constellation, developed independently by Chang Guang Satellite Technology Co., Ltd. (Changchun, China), has video, optical, and spectral imaging capabilities. The KF01A satellite is the core operational satellite, equipped with a 0.75 m panchromatic camera and a 3 m multispectral camera. The swath width of a single scene can reach 136 km, and it is widely used in urban construction, agricultural monitoring, ecological environment assessment, and geological disaster prevention, among other fields. The KF01A satellite imagery used in this study is fused from panchromatic and multispectral data, achieving a spatial resolution of 0.75 m, offering both high resolution and large coverage advantages. This imagery is selected as base map data to assist in the analysis of surface deformation characteristics in typical areas of the study region. The imagery can be accessed through the JL-1 satellite imagery data service platform (https://www.jl1mall.com/), accessed on 1 August 2025.

#### 2.2.3. Leveling Data

Leveling measurements are an important reference for verifying the accuracy of InSAR. Two leveling surveys were conducted during the study period: the first observation was from 25 February to 20 April 2023, with a midpoint on 24 March; the second observation was from 4 September to 31 October 2024, with a midpoint on 2 October. Through field observations, data processing, and height difference correction, the elevation change information for valid leveling points was obtained. The spatial distribution of the leveling points is shown in Figure 1.

### 2.3. Research Methods

This study utilizes 16 periods of Radarsat-2 SAR imagery data from 2022 to 2024. Based on the SBAS-InSAR method, the deformation rate and cumulative subsidence are inverted scene by scene. An InSAR mosaicking method is proposed, which uses the average value of the vertical overlapping area differences as a correction factor, to obtain a complete InSAR data product for urban surface subsidence monitoring.

#### 2.3.1. SBAS-InSAR Method

**Figure 2:**
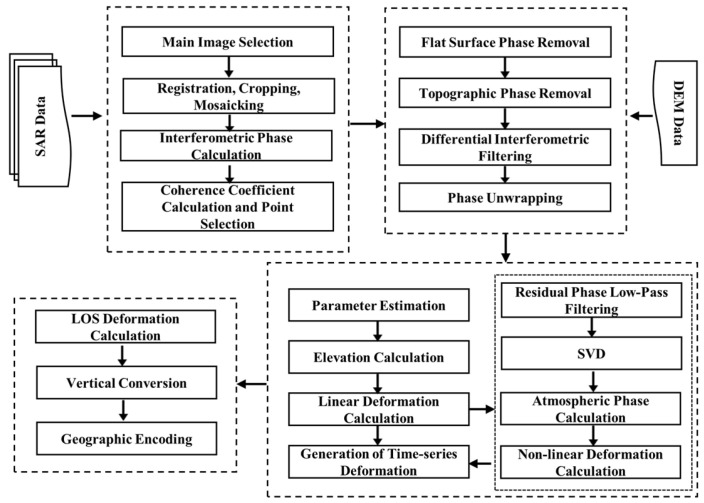
SBAS-InSAR processing flow.

InSAR is an active remote sensing monitoring technology that monitors ground deformation and change information by emitting and receiving electromagnetic waves. SBAS-InSAR, based on the small baseline subset interferometry principle, combines a large number of SAR images into subsets with short temporal and spatial baselines. This effectively alleviates the issue of temporal and spatial decorrelation caused by long baselines in traditional InSAR, improving the accuracy and reliability of surface deformation inversion [37]. The data processing steps are as follows: data preprocessing, selection of temporal and spatial baselines, small baseline interferogram generation, phase unwrapping, singular value decomposition, spatiotemporal filtering and atmospheric delay removal, reference point selection, deformation inversion, geographic encoding and projection transformation, and vertical deformation decomposition. The specific workflow is shown in Figure 2. In the SBAS-InSAR processing, GACOS data were incorporated to correct the interferograms for atmospheric delays, aiming to mitigate the influence of large-scale, slowly varying atmospheric phase delays on the phase observations. During the data processing, to ensure the quality of the interferometric phase, the coherence coefficient threshold of the interferogram is set to 0.3 [38]. The image registration accuracy is required to be better than 1/8 pixel in the azimuth direction to minimize the impact of geometric registration errors on the interferometric fringes and subsequent deformation inversion results [38].

#### 2.3.2. InSAR Mosaicking Method

The remote sensing image mosaicking is based on the matching of corresponding pixels in the overlapping regions, transforming multiple images to a unified spatial reference and adjusting the image gray values according to the gray level differences [24]. However, adjacent track InSAR results may exhibit discrepancies due to variations in observation geometry, atmospheric delay, reference point selection, and noise distribution. Direct mosaicking of the deformation results may introduce systematic biases. In this study, to achieve continuous monitoring of the study area, two different track SAR images were used for time-series InSAR analysis. After independently inverting each track, deformation results require calibration and mosaicking. Given that the differences between InSAR results in overlapping regions are mainly caused by geometric discrepancies between the tracks, the overall constant biases introduced by systematic errors, as well as random noise and inversion errors, a constraint-based correction method utilizing deformation differences in the overlapping region is proposed to unify the correction of multi-track InSAR results.

First, the original LOS deformation rates and cumulative displacements were converted into vertical components. During this conversion, horizontal motion components within the study area were assumed to be negligible; therefore, the LOS deformation was considered to predominantly reflect vertical motion. The mathematical expression is given as:

(1) $$V_{up}=\frac{V_{LOS}}{cos\theta }\;$$

where: *θ* is the incidence angle, *V_LOS_* is the deformation rate along the satellite line-of-sight, *cosθ* is the cosine of the incidence angle, and *V_up_* is the deformation rate in the vertical direction.

Using the vertical results of the corresponding coherent points in the overlapping region, the difference between the reference orbit and the orbit to be corrected is calculated, and the average value of this difference is taken as the correction factor, thereby achieving the calibration and mosaicking of InSAR results from adjacent orbits. The mathematical expression is as follows:

(2) $$\bar{∆V}=\frac{1}{N}\sum_{k=1}^{n}\left[V_{i}\left(x_{k},y_{k}\right)-V_{j}\left(x_{k},y_{k}\right)\right]\;$$

(3) $$V_{j}^{corr}\left(x,y\right)=V_{j}\left(x,y\right)+\bar{∆V}\;$$

where $V_{i}\left(x_{k},y_{k}\right)$ and $V_{j}\left(x_{k},y_{k}\right)$ are the subsidence rates of the k-th corresponding point in the overlapping area for the reference orbit and the orbit to be corrected, respectively. *N* is the number of overlapping points,$\;\bar{∆V}$ is the mean offset correction for the orbit to be corrected, $V_{j}^{corr}\left(x,y\right)$ is the corrected deformation result of the orbit to be adjusted.

To verify the rationality of approximating the inter-track differences as an overall constant offset, a statistical analysis of the deformation differences between the two InSAR tracks within the overlapping areas was conducted. The results indicate that, for each epoch, the deformation differences in the overlapping regions approximately follow a normal distribution, with a concentrated and symmetric main body and a small proportion of extreme outliers. The mean value is therefore able to effectively represent the overall offset introduced by imaging geometry differences and systematic errors. Accordingly, the mean deformation difference in the overlapping areas was adopted as the correction term to constrain and adjust the InSAR results of the track to be calibrated, thereby achieving consistency in the overall deformation level between inter-track datasets.

## 3. Results

### 3.1. Verification of the Inter-Orbit InSAR Mosaicking Method

**Figure 3:**
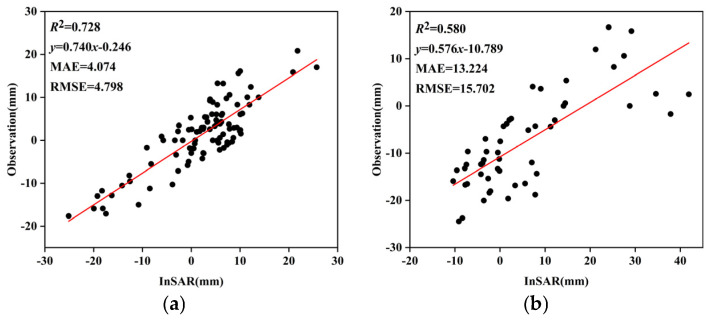
Validation of single-scene InSAR deformation results. (**a**) Track 1 validation; (**b**) Track 2 validation.

**Figure 4:**
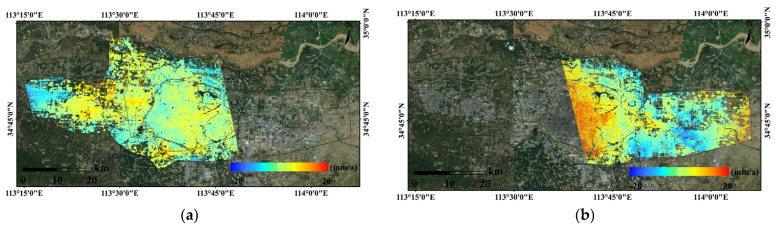
Average deformation rates of images 1 and 2. (**a**) Average deformation rates of images 1; (**b**) Average deformation rates of images 2.

SBAS-InSAR processing was performed independently on two stacks of Radarsat-2 SAR data, comprising a total of 16 scenes, to derive LOS deformation rates and cumulative displacements, which were further converted into vertical deformation components. Owing to the availability of only two epochs of leveling data and their incomplete temporal overlap with the InSAR observations, InSAR deformation results acquired at epochs closest in time to the leveling surveys were selected for comparative analysis, with the aim of evaluating the relative reliability of the InSAR-derived results from different tracks. Under these conditions, correlation analysis and regression were employed to assess the consistency between the InSAR results and ground-based measurements in terms of deformation trends and magnitudes. As shown in Figure 3a, the InSAR results from Track 1 exhibit a relatively strong correlation with the leveling data, with *R*^2^, MAE, and RMSE values of 0.728, 4.074, and 4.798, respectively, indicating a closer agreement in deformation magnitude. In contrast, Figure 3b shows that the results from Track 2 present a weaker correlation, with *R*^2^, MAE, and RMSE values of 0.580, 13.224, and 15.702, respectively; the validation points are more scattered and exhibit noticeable systematic bias. Furthermore, as illustrated by the single-track time-series deformation rates in Figure 4, Track 2 displays anomalously high uplift rates in the main urban area, which are inconsistent with the overall deformation characteristics of the study area. Based on this comprehensive analysis, Track 1 demonstrates higher consistency and stability in both the overall deformation pattern and magnitude. Accordingly, Track 1 was selected as the reference dataset, while Track 2 was treated as the dataset to be corrected for subsequent inter-track InSAR mosaicking and bias adjustment. Given the limited validation data and incomplete temporal overlap, this selection is primarily intended for relative calibration to optimize the performance of inter-track InSAR fusion.

**Figure 5:**
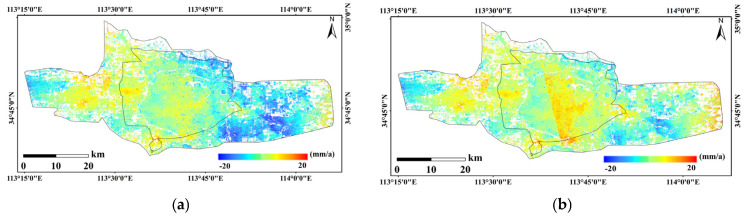
Map of average deformation rates. (**a**) Corrected deformation rate results; (**b**) Deformation rate results from direct mosaicking.

Using the deformation rate from two scenes of InSAR, corresponding coherent points in the overlapping region were extracted, and the deformation rate difference between the two orbits was calculated. The average deviation is approximately −5.54 mm/a. The average deviation was used to correct the data of the orbit to be matched, completing the unification of multi-orbit InSAR results. Figure 5a shows the corrected deformation rate results, while Figure 5b shows the deformation rate results from direct mosaicking. A comparison shows that the directly mosaicked deformation rate results exhibit significant seam effects, whereas the seam effect is nearly eliminated after applying the correction using the average value of the overlap region difference, with a smooth transition at the seam. From Figure 5a, it can be seen that the overall surface subsidence in the main urban area of Zhengzhou is relatively stable. The subsidence rate in the northeastern part of the main urban area is the most significant, reaching −15 mm/a. The central and western parts of the main urban area generally show uplift, with an average subsidence rate of around ±5 mm/a. An uplift condition is observed at the boundary between the main urban area and the western new city, while on the western side of the western new city, the overall subsidence rate is negative, with an average subsidence rate of about −10 mm/a. In the outer area of the main urban area and the eastern region, the subsidence rate is significant, with localized areas exceeding −15 mm/a, while the eastern new city’s eastern side remains relatively stable.

**Figure 6:**
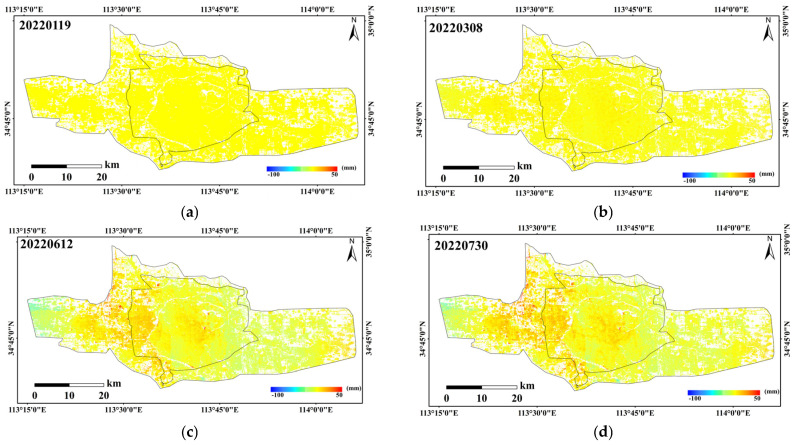

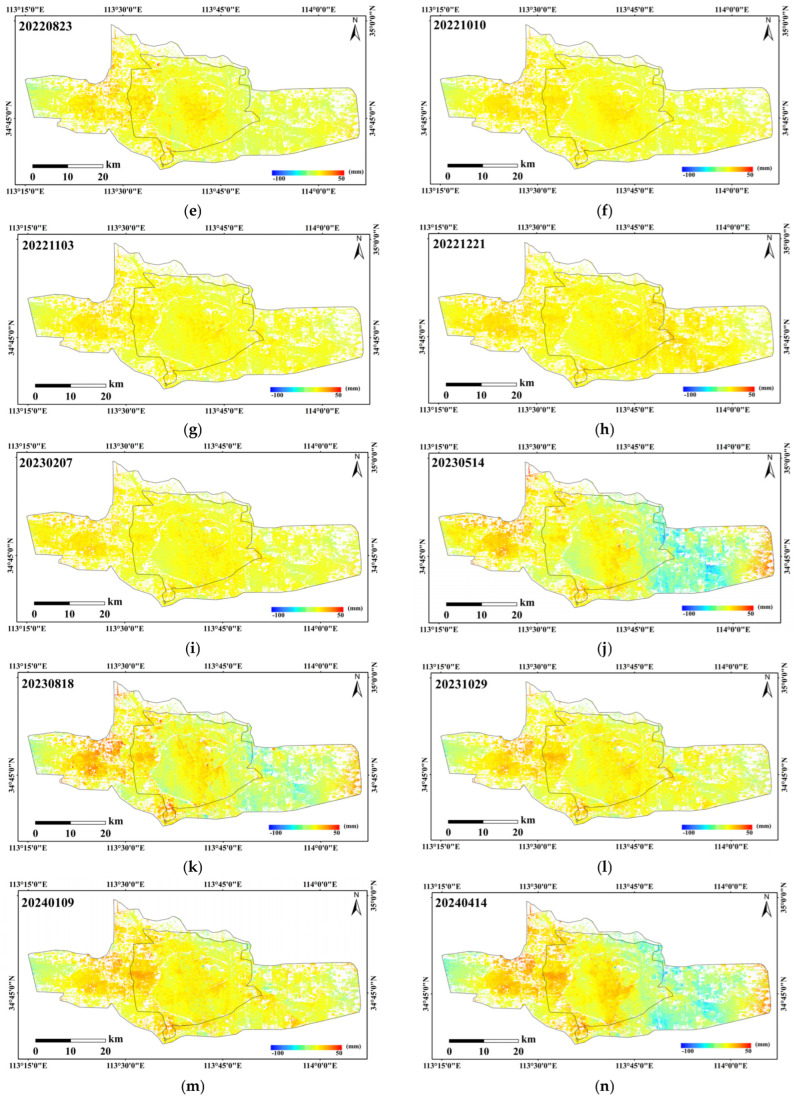

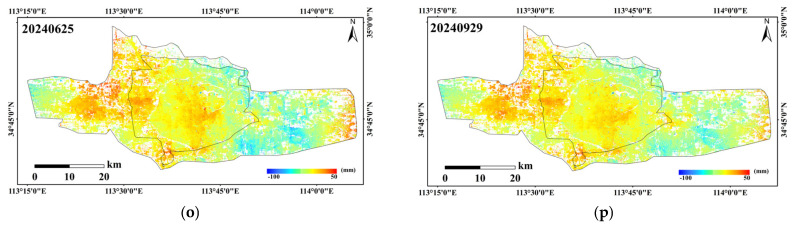
Cumulative deformation for each epoch from 2022 to 2024. (**a**) 19 January 2022; (**b**) 8 March 2022; (**c**) 12 June 2022; (**d**) 30 July 2022; (**e**) 23 August 2022; (**f**) 10 October 2022; (**g**) 3 November 2022; (**h**) 21 December 2022; (**i**) 7 February 2023; (**j**) 14 May 2023; (**k**) 18 August 2023; (**l**) 29 October 2023; (**m**) 9 January 2024; (**n**) 14 April 2024; (**o**) 25 June 2024; (**p**) 29 September 2024.

**Figure 7:**
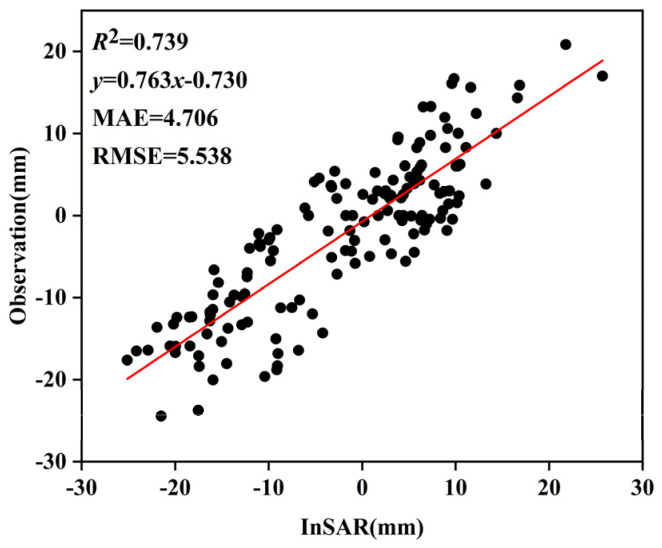
Validation using measured leveling points.

The unified correction method was applied to the cumulative subsidence values of each InSAR period. The correction values for each period’s InSAR results are shown in Table 2, and the corrected cumulative subsidence results are shown in Figure 6. The InSAR results and their fitting with the leveling points are shown in Figure 7. The results indicate that the corrected SBAS-InSAR results effectively reflect the variation trend of urban surface subsidence.

### 3.2. SBAS-InSAR Deformation Results Analysis

Through equidistant statistical analysis of the deformation rate and cumulative subsidence from InSAR inversion, the proportions of the average deformation rate and cumulative subsidence for the period 2022–2024 are shown in Table 3 and Table 4, respectively. From Table 3, it can be seen that the area exhibiting an uplift trend accounts for 37.6%, while the area exhibiting a subsidence trend accounts for 62.4%. Among these, the area with a deformation rate between 0~5 mm/a accounts for 28.64%, and the area with a deformation rate between −5~0 mm/a accounts for 30.33%. From Table 4, it can be seen that the area with cumulative subsidence accounts for 66.45%, while the area with cumulative uplift accounts for 33.55%. Among these, the area with a subsidence value between 0~±10 mm has the highest proportion, reaching 41.72%. Within the study area, there are still local regions with significant subsidence, with the maximum cumulative subsidence reaching approximately 100 mm.

### 3.3. Monitoring and Analysis of Key Subsurface Deformation Along Subway Lines

**Figure 8:**
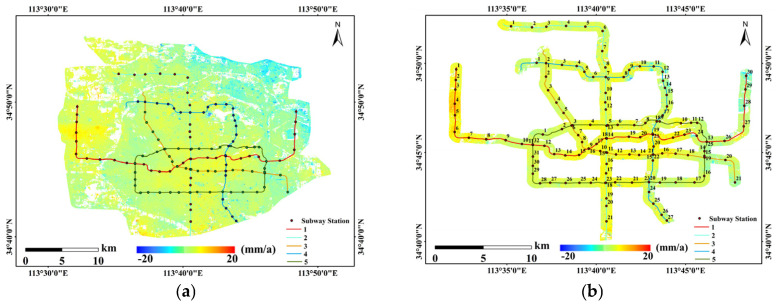
Metro line buffer zones and station coding map. (**a**) Subsidence rate map of the main urban area; (**b**) Buffer zone along the subway lines.

**Figure 9:**
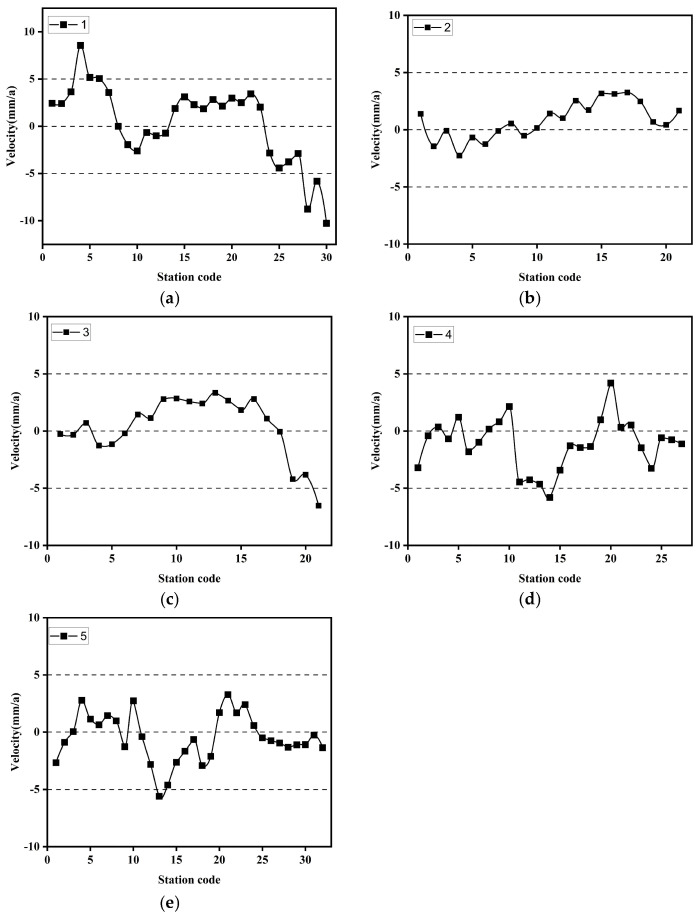
Deformation rates at metro line stations. (**a**) Line 1 station; (**b**) Line 2 station; (**c**) Line 3 station; (**d**) Line 4 station; (**e**) Line 5 station.

The subway lines are primarily distributed in the main urban area of Zhengzhou, where there are numerous high-rise buildings along the routes. This study established a 500-m buffer zone around the subway lines 1–5 in the main urban area (as shown in Figure 8, Figure 8a is the subsidence rate map of the main urban area, and Figure 8b shows the buffer zone along the subway lines). Based on this, the average deformation rate within the 500-m buffer zone of each station was calculated, and the statistical results are shown in Figure 9. From Figure 9a, it can be seen that the deformation rate at most of the stations along Line 1 is within ±5 mm/a, but there are still three stations where the deformation rate exceeds the ±5 mm/a reference line, with deformation rates reaching 8.75 mm/a, −8.76 mm/a, and −10.28 mm/a, respectively. The stations along Line 2 are generally stable, with the deformation rate fluctuating around ±3 mm/a. Most of the stations along Line 3 show uplift, with deformation rates within ±5 mm/a. However, from Dongzhou (Station 18) eastward, the deformation rate decreases significantly, with the largest decrease reaching −6.53 mm/a. Along Line 4, the deformation rate between stations 10 and 11 varies significantly, changing from 2.14 mm/a to −4.46 mm/a. Station 20 shows significant uplift, with a deformation rate of 4.19 mm/a. For Line 5, stations 13 and 14 have larger deformation rates, −5.6 mm/a and −4.61 mm/a, respectively, while other stations exhibit relatively stable deformation rates. In summary, the overall deformation rates of the stations along the five subway lines are within ±5 mm/a, with only the eastern section of Line 1 showing larger deformation rates. The query results show that the eastern part of Line 1, particularly the Longzihu area, is a reclaimed land area with soft soil layers in some sections. Moreover, since the Longzihu subway station opened in 2017, the region has not yet reached a stable subsidence phase. Additionally, the surrounding area, known as the University Town, includes numerous universities, residential, and commercial areas, which have increased the load in this region to some extent.

**Figure 10:**
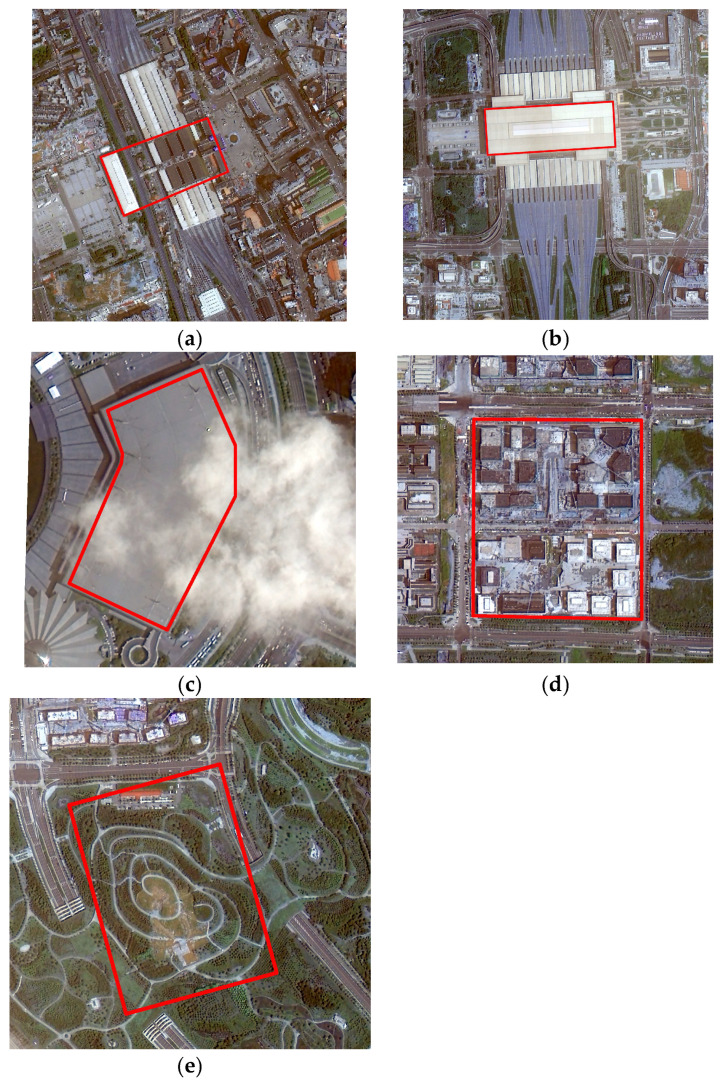
Map of key monitoring areas. (**a**) Zhengzhou Railway Station; (**b**) Zhengzhoudong Railway Station; (**c**) Convention and Exhibition Center; (**d**) Longhu Zhonghuan North construction area; (**e**) Yangjunliu Station.

**Figure 11:**
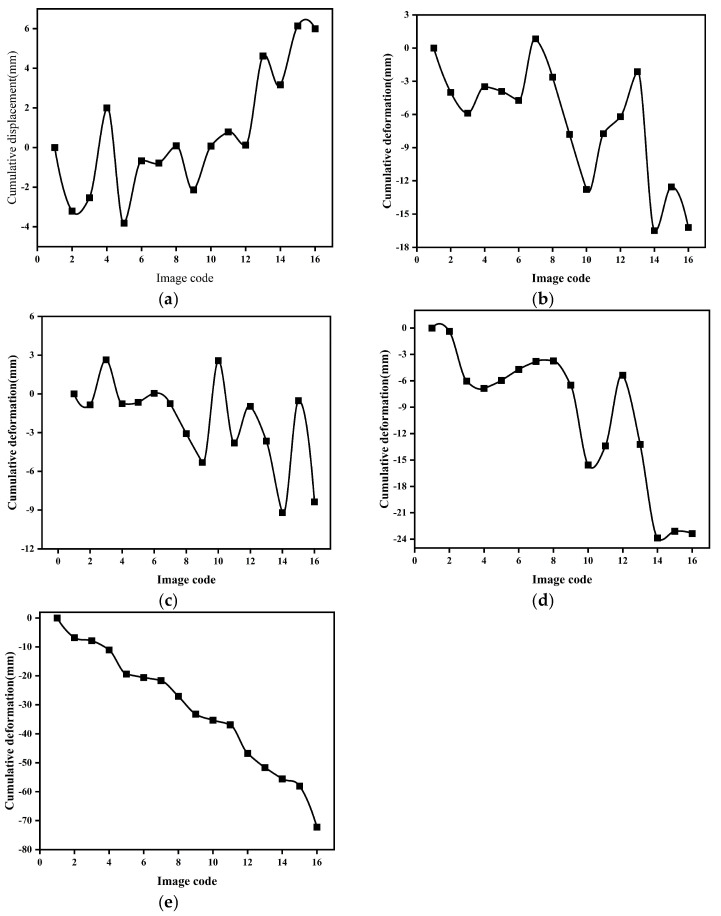
Cumulative subsidence in key monitoring areas. (**a**) Zhengzhou Railway Station; (**b**) Zhengzhoudong Railway Station; (**c**) Convention and Exhibition Center; (**d**) Longhu Zhonghuan North construction area; (**e**) Yangjunliu Station.

From the deformation rate results in the subway line buffer zones, it can be observed that there are significant subsidence funnels near some of the stations. To further analyze the subsidence situation, this study selected typical subsidence areas and important buildings along the subway lines using optical imagery for cumulative subsidence analysis. The selected buildings include Zhengzhou Railway Station and Zhengzhoudong Rail Station, located near Line 1 (as shown in Figure 10a,b). Typical subsidence areas include the Convention and Exhibition Center, the Longhu Zhonghuan North construction area, and the Yangjunliu Station along Line 4 (as shown in Figure 10c–e). For these five regions, vector polygons were drawn, and the cumulative subsidence amounts were calculated. The results are shown in Figure 11. The analysis results show that Zhengzhou Railway Station, located in the old town, exhibits a slight uplift trend overall. The other four regions, located in the eastern new district of the main urban area, show a continuous subsidence trend. Among these, the subsidence area near Yangjunliu Station is the most significant, showing a linearly accelerating trend, with a cumulative subsidence of approximately 80 mm from 2022 to 2024. Optical imagery verification revealed that this subsidence is closely related to the ongoing construction activities in the area.

## 4. Discussion

### 4.1. Deviation Analysis and Correction of Inter-Orbit InSAR Deformation Results

Currently, InSAR monitoring of urban surface subsidence typically relies on time-series solutions using single-orbit SAR imagery. However, for large-area coverage, multiple orbit data mosaicking is often required [39]. In this study, the two Radarsat-2 tracks have incidence angles of 39.57° and 30.55°, respectively. Differences in orbital geometry and acquisition time affect the sensitivity of the LOS measurements to vertical deformation and may also weaken the stability assumption of corresponding points within overlapping areas. To mitigate these effects, the LOS deformation results for each epoch were consistently transformed into vertical components, and a constrained correction based on the mean deformation differences of corresponding points in the overlapping areas was applied to optimize the multi-track mosaicking. During the conversion, horizontal deformation in the study area was assumed to be relatively small. Although this assumption may introduce some bias in the presence of significant horizontal motion, its impact on the overall analysis is limited, given that this study primarily focuses on inter-track consistency and relative correction.

Although GACOS data were introduced to correct tropospheric delays, the InSAR-derived deformation in this study is still obtained through relative estimation with respect to a reference point. Residual atmospheric delays, orbital errors, and uncertainties in reference point selection may therefore introduce a constant offset into the results, which can be further amplified during the fusion of multi-track InSAR datasets [39]. In addition, an imaging time difference between adjacent tracks increases the difficulty of maintaining consistency among multi-temporal results. To further quantify the overall constant offset potentially introduced by geometric differences and systematic errors, the deformation differences between the two InSAR tracks within the overlapping areas were statistically analyzed. The results show that, prior to correction, the deformation differences in the overlapping regions exhibit a pronounced mean offset. After applying a constrained correction based on the mean deformation differences at corresponding points within the overlapping areas, the mean of the difference distribution is significantly reduced, and the deformation estimates from different tracks within the overlapping regions converge to a consistent overall level. These findings demonstrate that the constrained correction method based on the statistical characteristics of overlapping areas can effectively mitigate the overall constant offset induced by orbital geometry differences and systematic errors, thereby improving the consistency of inter-track InSAR mosaicking results. The methodology employed in this study represents a relatively simplified processing approach, which may be insufficient to fully characterize the spatial heterogeneity of errors. When orbital errors or residual atmospheric effects exhibit significant spatial trends, correction using a single constant term may be limited. Nevertheless, in this study, the seam effects in the deformation field after correction based on overlapping regions were significantly reduced, the transitions between stitched tracks were smooth, and the statistical metrics compared with leveling observations were improved. These results indicate that the proposed approach is applicable under the spatial scale and data conditions of the study area. Future research could further incorporate spatial trend modeling or more sophisticated error correction schemes to enhance the robustness and accuracy of multi-track InSAR fusion results.

As only two epochs of leveling measurements were available in this study, and their acquisition times did not fully coincide with those of the InSAR data, the validation accuracy and reliability may be affected. Surface deformation generally exhibits nonlinear behavior, which further constrains the absolute calibration of the InSAR-derived deformation field. Future studies could incorporate additional leveling and GNSS observations, particularly validation data with higher temporal frequency and denser spatial coverage, to improve the accuracy and reliability of the monitoring results.

### 4.2. Spatiotemporal Characteristics of Urban Surface Subsidence

Previous studies have shown that the causes of surface uplift can be broadly classified into two categories: (1) crustal movement leading to surface elevation changes; (2) foundation rebound effects caused by groundwater level rise. Zhengzhou’s overall topography slopes from the southwest to the northeast, with the city mainly distributed in the Yellow River alluvial plain, where the strata consist primarily of loose sediments that are highly compressible and easily deformed by external disturbances. Analysis indicates that if crustal movement dominates, it usually results in widespread uplift or subsidence. However, the subsidence rate map of Zhengzhou shows that uplift is mainly concentrated within the city’s Fourth Ring Road, with uplift rates lower than 10 mm/a, and the overall subsidence is relatively gentle. Therefore, the surface subsidence in the main urban area of Zhengzhou is attributed to groundwater extraction. Huang et al. analyzed ground subsidence along Zhengzhou’s subway lines using Sentinel-1 data and found that most areas along the subway line exhibited small deformation rates [40], consistent with the results of this study. Further research shows that Zhengzhou has implemented a groundwater ban and restriction policy in the main urban area since 2015 [41]. The uplift area corresponds closely with the coverage of this policy, further verifying that the uplift is closely related to the rise in groundwater levels. Ding and Wang’s research indicates that groundwater dynamics and subsidence variations are highly consistent over time [41,42], confirming the reliability of attributing surface uplift to the rise in groundwater levels and the foundation rebound effect of the Quaternary sand and gravel layers after water absorption. In contrast to the main urban area, surface subsidence in the eastern new districts is more significant. This is mainly due to the loose soil layers in the area, rapid urbanization, and excessive groundwater extraction. Large-scale construction of transportation infrastructure, residential, and commercial areas, along with localized over-extraction of groundwater, has caused the subsidence rate in this area to generally be higher than in the main urban area. Wang et al. conducted a PS-InSAR experiment in Zhengzhou using Envisat-1 data from 2004 to 2010 and found that the major surface subsidence areas in Zhengzhou are concentrated in the northern and eastern parts of the city [43]. This finding is consistent with the results of this study, indicating that the trend of surface subsidence in the main urban area of Zhengzhou has not changed significantly over time. Surface subsidence in the western new district is relatively stable, mainly due to its proximity to the Songshan and Jishan mountain areas, where the terrain is higher, and the foundation conditions are better. Time-series InSAR results from typical areas show that different building groups exhibit significantly different subsidence responses. Localized subsidence is not only controlled by fluctuations in groundwater levels but also influenced by building structural characteristics, load differences, and surrounding construction activities. Overall, surface subsidence in Zhengzhou shows a trend of uplift in the west and subsidence in the east. The main urban area is relatively stable, while the surrounding new districts and northeastern regions exhibit more significant subsidence.

### 4.3. Attribution Analysis of Subsidence Along Subway Lines

Existing urban surface subsidence monitoring studies often focus on overall subsidence patterns, with relatively fewer discussions on the specific subsidence characteristics and causes along subway lines. In this study, by establishing buffer zones along the subway lines for analysis, it was found that even though there is an overall slight uplift trend in the main urban area, significant subsidence anomalies still exist along the subway lines, as shown in Figure 8b. A comparative analysis of typical areas shows that the deformation around Zhengzhou Railway Station is relatively small, with overall subsidence tending to stabilize. This is mainly due to the early operation of the railway station and Metro Line 1, with the strata gradually consolidating and stabilizing under long-term load and disturbance. Relevant studies have also indicated that subway lines generally enter a stable phase after approximately ten years of operation. In contrast, subsidence around several stations in the eastern new district of the main urban area is more significant. This is primarily due to the area’s geological conditions, which are dominated by loose sediments with relatively poor stability. Additionally, the combined effect of subway tunnel excavation and high-rise construction has exacerbated surface subsidence, showing a clear downward trend. It should be noted that there is currently a lack of detailed geological profiles and groundwater dynamic monitoring data covering the areas around subway stations, which results in some uncertainty regarding the geological causes of subsidence along subway lines. Future studies should incorporate multi-source data and numerical simulations for further in-depth verification.

## 5. Conclusions

Based on two stacks comprising 16 Radarsat-2 SAR scenes acquired during 2022–2024, this study employed the SBAS-InSAR technique to monitor surface subsidence and subway-line infrastructure in Zhengzhou City. By introducing a constrained mosaicking strategy using the mean deformation differences of corresponding points within overlapping areas, a spatially continuous deformation field was constructed, achieving consistency between inter-track InSAR results. The main conclusions are summarized as follows:

(1) After mosaicking and correction, the InSAR results show good agreement with leveling measurements, with *R*^2^ = 0.739, MAE = 4.706 mm, and RMSE = 5.538 mm, indicating satisfactory consistency at the relative deformation level. (2) Surface subsidence in the main urban area of Zhengzhou is generally stable, with deformation rates predominantly within ±5 mm/a, while slightly higher subsidence rates are observed in the eastern new district. (3) Along the subway lines, relatively higher subsidence rates are detected in the eastern section of Metro Line 1, particularly around the Longzihu area, whereas other lines remain largely stable, with deformation rates mostly within ±5 mm/a.

Through the analysis of the corrected two-track InSAR mosaicking results, this study provides reliable relative deformation reference data for urban infrastructure monitoring, especially along subway corridors. Future work may incorporate additional multi-temporal SAR imagery as well as denser leveling and GNSS observations to further enhance the accuracy and reliability of subsidence monitoring.

## Figures and Tables

**Table 1:** Acquisition dates of SAR imagery.

No.	Image 1	Image 2	No.	Image 1	Image 2
1	20220119	20220202	9	20230207	20230221
2	20220308	20220322	10	20230514	20230621
3	20220612	20220602	11	20230818	20230808
4	20220730	20220720	12	20231029	20231019
5	20220823	20220906	13	20240109	20240123
6	20221010	20220930	14	20240414	20240428
7	20221103	20221024	15	20240625	20240709
8	20221221	20221211	16	20240929	20241013

**Table 2:** Correction values of cumulative InSAR deformation for each epoch in Track 2.

No.	Correction Value (mm)	No.	Correction Value (mm)
1	0	9	−4.77
2	−3.65	10	−27.44
3	−13.89	11	−15.18
4	−10.56	12	−7.07
5	−10.69	13	−1.26
6	−7.05	14	−22.63
7	−3.78	15	−22
8	1.65	16	−17.13

**Table 3:** Statistical distribution of deformation rate by area (%).

No.	Range of Subsidence Rates (mm/a)	Proportion
1	15~20	0.11%
2	10~15	1.21%
3	5~10	7.64%
4	0~5	28.64%
5	0~−5	30.33%
6	−10~−5	19.80%
7	−15~−10	9.50%
8	−20~−10	2.77%

**Table 4:** Statistical distribution of cumulative subsidence by area (2022–2024) (%).

No.	Range of Subsidence Amounts (mm)	Proportion
1	50~40	0.44%
2	40~30	0.76%
3	30~20	2.94%
4	20~10	9.92%
5	10~0	19.49%
6	0~−10	23.00%
7	−10~−20	18.72%
8	−20~−30	13.12%
9	−30~−40	7.21%
10	−40~−50	2.96%
11	−50~−100	1.42%
12	−100~−150	0.02%

## Data Availability

The data used in this study is available by contacting the corresponding author.
